# ﻿Discovery and lectotype designation of *Longitarsuscalifornicus* (Motschulsky) (Coleoptera, Chrysomelidae, Galerucinae, Alticini)

**DOI:** 10.3897/zookeys.1209.124692

**Published:** 2024-08-09

**Authors:** Alexander S. Konstantinov, Vladimir Yu. Savitsky, Il’ya A. Zabaluev

**Affiliations:** 1 Systematic Entomology Laboratory, USDA, ARS, c/o Smithsonian Institution, National Museum of Natural History, Washington DC, USA National Museum of Natural History Washington DC United States of America; 2 Zoological Museum of Lomonosov Moscow State University, Moscow, 125009, Russia Moscow State University Moscow Russia

**Keywords:** America north of Mexico, Il’ya Gavrilovich Voznesensky, Johann Friedrich Gustav von Eschscholtz, key for identification, leaf beetles

## Abstract

The lectotype of *Longitarsuscalifornicus* (Motschulsky, 1845) is designated, described, and illustrated. An illustrated key to eight light-colored *Longitarsus* species known to occur in the western United States is presented. A brief history of Russian entomological collecting in North America during the first half of 19^th^ century, with specimens preserved in Zoological Museum of Moscow University, Moscow and Zoological Institute, St. Petersburg, is provided.

## ﻿Introduction

*Longitarsus* Latreille, 1829 is the most species-rich genus among flea beetles, with more than 700 species worldwide (Konstantinov unpublished compilation). Fifty-one valid species of *Longitarsus* are known to occur in America north of Mexico (Riley et al. 2003; Konstantinov unpublished compilation). Thirty-nine of them are native, and 12 are introduced, either as biological control agents of invasive weeds or unintentionally (LeSage 1988; Pentinsaari et al. 2019). North American *Longitarsus* has never been reviewed or revised. The most recent available key for species identification is that of Horn (1889). A few regional keys were later published, which included *Longitarsus* (e.g. Beller and Hatch 1932; Wilcox 1954; Balsbaugh and Hays 1972). Most North American *Longitarsus* species were described by Blatchley (1923), Horn (1889), and LeConte (1859), and their type specimens are available for study at major Canadian and United States entomological collections (Canadian National Collection, Ottawa, Ontario; Museum of Comparative Zoology, Cambridge, Massachusetts; Purdue Entomological Research Collection, West Lafayette, Indiana; and National Museum of Natural History, Washington DC). However, the whereabouts of the type specimen and, therefore, the certain identity of one species, *Longitarsuscalifornicus* (Motschulsky, 1845), has remained a mystery until recently, when a single female specimen was discovered by VYS in one of the drawers of the Motschulsky collection in the Zoological Museum of Moscow University, Moscow, Russia (ZMUM). The Motschulsky beetle collection contains approximately 60,000 specimens, of which about 4,000 are types (Lyubarsky 2009). Most of the types have been recognized as such and transferred from the main holdings to special drawers. However, beetle types previously considered missing are still being discovered in the main holdings of Motschulsky collection (Savitsky 2018). The *Longitarsuscalifornicus* specimen was pinned on a short pin with a small label in such a way that the specimen was close to the bottom of the drawer and was not recognized as the type by previous ZMUM curators.

The origin of the specimen and its exact collecting locality remain unknown. It could not have come from Motschulsky’s own collecting in the United States, which he visited in 1853–1854, nearly 10 years after the description of *L.californicus* was published. In a letter to Édouard Ménétries dated July 15, 1854, Motschulsky mentioned a visit to the LeConte collection which contained “very different things than what Dr. Voznesensky brought” from California (Motschulsky 2013). Indeed, there is a slight possibility that the specimen of *L.californicus* came from Il’ya Gavrilovich Voznesensky (1816–1871), who travelled to western North America in 1839–1849 (Gnucheva 1940), where he visited Fort Ross, Drake’s Cape, San Francisco Bay, Fort Ross at Bodega Bay, Mount St. Helens, and Khlebnikov Valley. Each location originally had its own color-coded label (Feklova 2014). However, Motschulsky often replaced original labels with his own (Koponen and Niemelä 2020), so the color of the *L.californicus* label cannot help in identifying its type locality. The bulk of the Voznesensky zoological collection was transferred to the Zoological Institute (St. Petersburg, Russia) most likely around 1849 (Feklova 2014).

The other likely source of California specimens described by Motschulsky in 1845 is collection of Johann Friedrich Gustav von Eschscholtz (1793–1831), an early pioneer in the western North American coleopterology. He was the naturalist in two expeditions in 1815–1817 and 1823–1826 to the western United States (Koponen and Niemelä 2020). Beetles are well represented among large biological collections that he made. New beetle genera and species were described by Eschscholtz (1829–1833) and Mannerheim (1843) based on North American specimens collected by Eschscholtz. Eschscholtz’s collection was transferred to the Zoological Museum of Moscow University in the summer of 1837 (Lyubarsky 2009) at a time of Motschulsky’s affiliation with the museum and well before 1845 (Koponen and Niemelä 2020).

## ﻿Materials and methods

The source of the flea beetle diversity is an unpublished compilation of flea beetle genera and species of the world, which is a FileMakerPro database maintained by ASK since 2006. It is cited as “Konstantinov unpublished compilation”. The lectotype of *L.californicus* was processed as follows. The abdomen, genitalia and terminalia were studied at magnifications up to ×400 (spermatheca) and documented from glycerol preparations, using a Micromed-3 microscope equipped with a ToupCam 9.0 MP digital eyepiece camera. The other photographs were taken using a Canon EOS 5D Mark IV camera with a Canon MP-E 65 mm objective lens. The USNM specimen of *L.californicus* was photographed with Macropod Pro photomacrography system (Macroscopic Solutions, LLC, Tolland, CT, USA) and processed with Zerene Stacker v. 1.04 and edited with Adobe Photoshop Elements 2020. Dissecting techniques and morphological terminology follow Konstantinov (1998). Numbering only visible tarsomeres and abdominal segmentation justified previously (Konstantinov et al. 2022). The type locality is cited verbatim as it appears in the original description.

Specimens studied in this paper are deposited in the following collections:

**USMN**National Museum of Natural History, Washington DC, USA.

**ZMUM**Zoological Museum of Moscow University, Moscow, Russia.

## ﻿Results

### ﻿ Longitarsus californicus

Taxon classificationAnimaliaColeopteraChrysomelidae

(Motschulsky)

884711AB-0E95-5B9A-8255-00A0D07840DA

Figs 1–8 , 9–15 , 16–23

Teinodactyla californica Motschulsky, 1845: 382 (type locality: Californie; lectotype, ♀, designated here, ZMUM).

#### Type material examined.

***Lectotype***: ♀, labels (Figs 7, 8) (ZMUM).

(1) “Teinodact californica m California” in V.I. Motschulsky’s handwriting on white paper;

(2) “Zoomuseum of MSU (Moscow, RUSSIA) [in Russian] Nº ZMUM Col 02777 Zool. Mus. Mosq. Univ. (Mosquae, ROSSIA) ex coll. V. I. Motschulsky” printed on pink paper;

(3) “ Lectotypus *Teinodactylacalifornica* Motschulsky, 1845 A. Konstantinov, V. Savitsky et I. Zabaluev des. 2024” printed on red paper;

(4) “ *Longitarsuscalifornicus* (Motschulsky, 1845) A. Konstantinov det. 2024” printed on white paper.

Lectotype is missing hind right leg, left protarsomeres 2–4, and 11 antennomere of right antenna. Antennomeres 3–11 of left antenna, left protarsomere 1, right mesotibia and mesotarsi are glued to a white card below the specimen mount. Abdomen and genitalia are placed in genitalia vial with glycerin.

#### Material examined.

(2 ♀, 1 ♂, USNM).

(1) “Amedee, Cal, July 21–28, 4200 ft, Wickham”;

(2) “Wickham Collection, 1933”;

(3) “*Longitarsuscalifornicus* Horn” handwritten on yellowed paper with a red border;

(4) “*Longitarsuscalifornicus* (Motschulsky), det A. Konstantinov 2024”.

#### Diagnosis.

Head with vertex covered by reticulation. Supracallinal sulci thin, antennal calli separated from vertex by thin line. Frontal ridge elongate, wider between antennal sockets, narrower towards clypeus. Antennomere 2 longer than 3. Antennomere 3 as long as 4. Pronotal surface with coarse reticulation. Elytra posteriorly about as long as abdomen, covering nearly all abdominal tergites. Receptacle of spermatheca elongate. Receptacle and pump distinctly to abruptly separated from each other. Spermathecal canal with coils. Vaginal palpus slender, with apex subdeltoid. Anterior sclerotization of vaginal palpus much narrower than posterior sclerotization.

#### Description.

**Figures 1–8.**
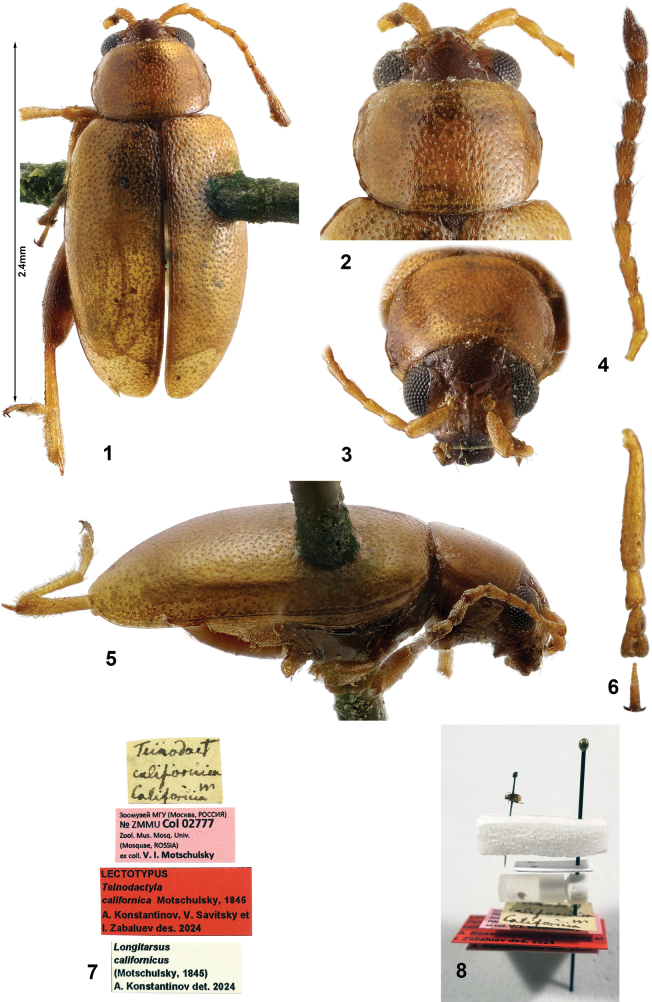
*Longitarsuscalifornicus* (Motschulsky), Lectotype **1** habitus, dorsal view **2** pronotum **3** head, frontal view **4** left antennae with antennomeres 1 and 2 missing **5** habitus lateral view **6** right mesotibia and mesotarsi **7** labels **8** lectotype as currently mounted with labels and genitalia vial.

***Body*** (Figs 1, 5) length 2.21–2.40 mm, width 1.08–1.15 mm (the lectotype 2.4 mm long and 1.15 mm wide). Pronotum and elytra light yellowish; 5 apical antennomeres, head and metafemur slightly darker.

**Figures 9–15.**
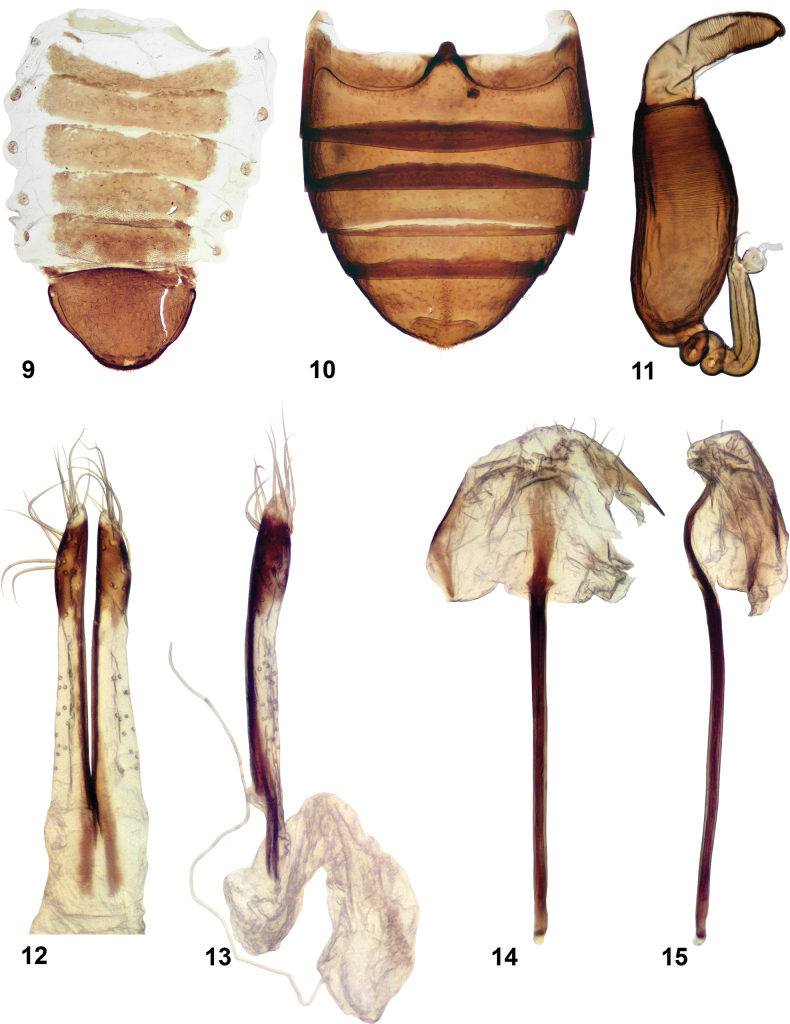
*Longitarsuscalifornicus* (Motschulsky), Lectotype **9** abdominal tergites **10** abdominal ventrites **11** spermatheca **12, 13** vaginal palpi, ventral and lateral views **14, 15** tignum, ventral and lateral views.

**Figures 16–23.**
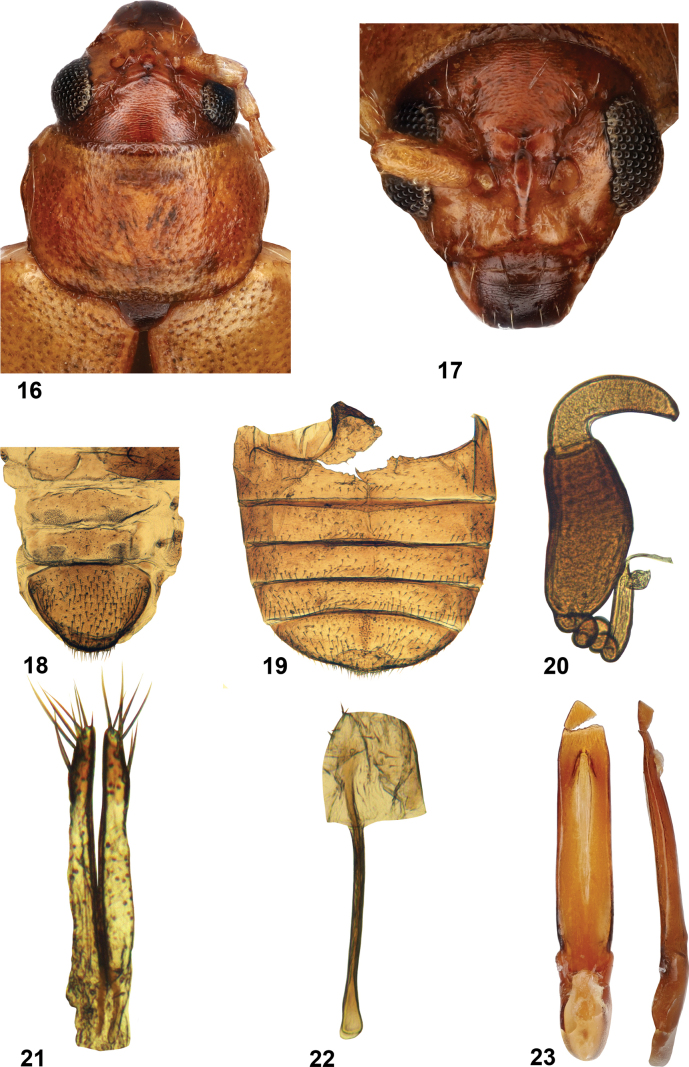
*Longitarsuscalifornicus* (Motschulsky), California specimens (USNM) **16** pronotum and head dorsal view **17** head, frontal view **18** abdominal tergites **19** abdominal ventrites **20** spermatheca **21** vaginal palpi, ventral view **22** tignum, ventral view **23** median lobe of aedeagus, ventral and lateral views.

***Head*** (Figs 2, 3, 16, 17). Vertex covered with relatively coarse reticulation. Supracallinal sulci thin, antennal calli separated from vertex by thin line. Surface of antennal calli moderately shiny, lacking reticulation, but with few minute punctures. Frontal ridge elongate, wider between antennal sockets, narrower towards clypeus. Anterofrontal ridge relatively narrow (Figs 3, 17), posteriorly gradually merging with frons, forming inverted T-shaped structure with frontal ridge. Antennomere 2 longer than 3. Antennomere 3 about as long as 4.

***Thorax.*** Pronotal punctures relatively large (Figs 2, 16), slightly smaller than elytral punctures, as densely placed as elytral punctures. Surface between punctures coarsely reticulated. Elytra with humeral calli well developed. Elytral punctures do not form longitudinal rows. Surface between punctures reticulated. Female pro- and mesotarsomere 1 as wide as pro- and mesotarsomere 2. In males protarsomere 1 about twice as wide as protarsomere 2; mesotarsomere 1 wider at the base narrowing towards apex.

***Abdomen*** (Figs 9, 10, 18, 19). In female, abdominal tergites 5 and 6 with two symmetrically placed patches of short setae. Pygidium with evenly spaced long setae. Abdominal ventrite 2 with marginal setae interrupted on both sides of the middle (Fig. 19). Complete rows of marginal setae situated on ventrites 3 and 4. Ventrite 5 with middle strip lacking long setae.

***Genitalia*** (Figs 11–15, 20–23). Receptacle of spermatheca elongate, distinctly separated from pump, much longer than it. Internal side convex, external side concave. Pump with short curved denticle on top. Spermathecal canal with multiple coils, at base directed along the side of receptacle. Vaginal palpus slender with apex subdeltoid. Anterior sclerotization of vaginal palpus much narrower than posterior sclerotization. Tignum with posterior sclerotization about as wide as middle. Anterior sclerotization variable; in lectotype narrow, not wider than middle; in USNM specimen wider than middle, spoon-shaped. Median lobe of aedeagus nearly straight in lateral view, apex slightly S-shaped. In ventral view nearly parallel-sided. Apex gradually narrowing, without denticle. Membranous window narrow, constricting towards base and not reaching it.

## ﻿Discussion

In addition to the female lectotype, we studied three other specimens, two females and one male, identified as *L.californicus* (USNM). The identification label for these does not have the name of the identifier, and we could not recognize the handwriting, so we do not know who made that identification. The identification label lists Horn as the author of the species; however, we could not find any *Longitarsus* named *californicus* by Horn. Dissection of one female revealed that the genitalia, especially the spermatheca and vaginal palpi, are very similar to those of the lectotype of *L.californicus*. The tignum of the lectotype (Fig. 14) is slightly different from that of the USNM specimen (Fig. 22) in having a narrower anterior part. In other features, the lectotype and female USNM specimens are very similar. Therefore, we confirm the identification of the three USNM specimens, including the male, as *L.californicus*.

Eight yellow *Longitarsus* species are known to occur in the western United States, as delimited by Lingafelter (2007) and Yanega (1996). Two species, *L.jacobaeae* Waterhouse and *L.flavicornis* Stephens, were introduced into North America (LeSage 1988). As shown in previous studies, *Longitarsus* species may be sorted into species groups based on the general shape of their median lobe of aedeagi (Konstantinov and Dorr 2023; Liang et al. 2023). *Longitarsuscalifornicus* is clearly close to *L.livens* LeConte and *L.vanus* Horn in having the median lobe nearly straight in lateral view and with only a slightly S-shaped apex. In ventral view, the lobe is nearly parallel-sided but slightly narrower in the middle. The apex is gradually narrowing and without a well-differentiated denticle. The membranous window is narrow and constricted towards base but not reaching it (Fig. 23). *Longitarsuscalifornicus* can be separated from these and other light-colored *Longitarsus* species known to occur in western United States using the following key.

### ﻿Preliminary illustrated key to yellow *Longitarsus* species occurring in the western United States

Some species in this sample are represented by only a single male or female, and, therefore, in it is impossible to use characters of genitalia in some parts of the key.

**Table:** 

1	Antennomere 2 longer than 3	**2**
–	Antennomere 2 as long as or shorter than 3	**4**
2	Spermatheca with canal not extending away from receptacle and runs parallel to it at base. Spermathecal pump much shorter and narrower than receptacle. Median lobe of aedeagus nearly parallel-sided in ventral view	***L.californicus* (Motschulsky)**
–	Spermatheca with canal extending away from receptacle and runs away from it at base. Spermathecal pump about as long as receptacle. Median lobe of aedeagus more or less constricted in ventral view	**3**
3	Horizontal part of spermathecal pump merging with vertical part. Median lobe of aedeagus less constricted more or less parallel sided in ventral view. Apex more elongate.	***L.jacobaeae* Waterhouse**
–	Horizontal and vertical parts of spermathecal pump with distinct border. Median lobe of aedeagus more constricted in ventral view. Apex rounder.	***L.flavicornis* (Stephens)**
4	Supracallinal sulci absent, antennal calli at times make fold with vertex	**5**
–	Supracallinal sulci thin, but present, antennal calli separated from vertex by thin line	**7**
5	Lateral sides of aedeagus in ventral view constricted before apical one-third	***L.flavicornis* (Stephens)**
–	Lateral sides of aedeagus in ventral view converging from base to apex, nearly straight	**6**
6	Median lobe of aedeagus in lateral view nearly straight before apical one-quarter, in ventral view apex less acute	***L.livens* LeConte**
–	Median lobe of aedeagus in lateral view bends dorsally and then ventrally before apical one-quarter, in ventral view apex more acute	***L.vanus* Horn**
7	Pronotal punctures small, sharply impressed	***L.flavicornis* (Stephens)**
–	Pronotal punctures comparatively larger, less sharply impressed	**8**
8	Head nearly same color as pronotum	***L.subrufus* LeConte**
–	Head darker in color than pronotum	**9**
9	Vertex covered with coarse, deeply impressed reticulation	***L.repandus* LeConte**
–	Vertex covered with fine, shallowly impressed reticulation	***L.occidentalis* Horn**

## Supplementary Material

XML Treatment for Longitarsus californicus

## ﻿Additional information

### Author ORCIDs

Alexander S. Konstantinov https://orcid.org/0000-0001-6578-6735

Vladimir Yu. Savitsky https://orcid.org/0000-0002-6680-754X

Il’ya A. Zabaluev https://orcid.org/0000-0002-1558-5502

### Data availability

All of the data that support the findings of this study are available in the main text.
